# Butyrate down-regulates CD44 transcription and liver colonisation in a highly metastatic human colon carcinoma cell line

**DOI:** 10.1038/sj.bjc.6600574

**Published:** 2002-11-12

**Authors:** M Barshishat, I Levi, D Benharroch, B Schwartz

**Affiliations:** Institute of Biochemistry, Food Science and Nutrition, Faculty of Agricultural, Food and Environmental Quality Sciences, The Hebrew University of Jerusalem, Rehovot 76100 Israel; Surgery Department, Soroka University Hospital of Kupat-Holim, the Faculty of Health Sciences, Ben-Gurion University of the Negev, Beer-Sheva, Israel; Pathology Department, Soroka University Hospital of Kupat-Holim, the Faculty of Health Sciences, Ben-Gurion University of the Negev, Beer-Sheva, Israel

**Keywords:** CD44, butyrate, colorectal cancer, metastasis, extracellular matrix

## Abstract

Over-expression of the adhesion molecule CD44 and its splice variants, especially CD44v6, is associated with poor prognosis and metastasis. We aimed at regulating the expression of CD44 in the highly metastatic human colon cancer cell line HM7 and thereby affecting its metastatic ability. HM7 cells show constitutive expression of CD44 standard and variants isoforms, which were significantly down-regulated by treatment with butyrate. Butyrate significantly inhibited transcription of the CD44 gene and abolished epidermal growth factor-mediated up-regulation of the reporter gene luciferase subcloned upstream to the CD44 promoter (−1.1 kb) and transfected to HM7 cells. Nuclear proteins from butyrate-treated cells bound to an epidermal growth factor receptor element motif present in the CD44 promoter. Epidermal growth factor receptor element-site directed mutations eliminated the inducibility of the luciferase reporter gene and did not allowed binding of nuclear proteins harvested from butyrate-treated cells. Butyrate induced CD44 gene repression by specifically interacting with an epidermal growth factor receptor element nuclear transcriptional factor. This interaction affects CD44 transcriptional activity vis-à-vis *in vivo* metastatic ability of HM7 cells. These results provide additional insight into the anticarcinogenic properties of butyrate.

*British Journal of Cancer* (2002) **87**, 1314–1320. doi:10.1038/sj.bjc.6600574 www.bjcancer.com

© 2002 Cancer Research UK

## 

Adhesion molecules expressed on metastatic or disseminated cells are indispensable for the cell's transmigration through the endothelium, locomotion in the blood-lymph circulation, and localization in the target organ (Lester and Maccarthy, 1992). Homotypic and heterotypic, cell-to-cell and cell-to-matrix interactions are involved in each of these steps (Herrera-Gayol and Jothy, 1999). CD44 is one of the few proteins able to fulfil this dual role. CD44 comprises a family of many glycoprotein isoforms (Rudzki and Jothy, 1997). The CD44 variant isoforms differ from the standard form, CD44s, in their proximal extracellular region. There, 10 exons, designated v1 to v10, can be inserted by alternative splicing (Screaton *et al*, 1992). CD44 is a widely distributed cell-adhesion molecule that has been implicated in the metastasis of lymphomas and epithelial tumours, such as colorectal cancer. In human cancer, CD44 splice variants are frequently expressed in advanced stages of tumorigenesis. Structural analysis of the CD44 upstream regulatory region in human neuroblastoma cells revealed that there is a cis-regulatory element that contributes to downregulation of the CD44 gene. This epidermal growth factor (EGF) regulatory element (ERE) sequence of CD44 (Shtivelman and Bishop, 1991), unlike other cis-acting elements that contain the Sp1 site, comprises a specifically oriented 22-bp sequence, necessary to enable EGF-induced CD44 gene transcription through a specific interaction between the ERE motif and an unknown transcription factor. Point mutations within this sequence alter CD44 transcription (Zhang *et al*, 1997).

Butyrate is a short-chain fatty acid formed in the gastrointestinal tract of mammals as a result of anaerobic bacterial fermentation of undigested dietary components, and is avidly absorbed by the colonic epithelium. Treatment of mammalian cells *in vitro* with millimolar concentrations of butyrate has pleiotropic effects on cellular physiology (Hassing *et al*, 1997; Schwartz *et al*, 1998; Barshishat *et al*, 2000). Butyrate exerts several effects on nuclear proteins that could modify gene expression (Nakano *et al*, 1997; Archer *et al*, 1998). The aim of the present study was to assess whether CD44 can be regulated in high metastatic colon cancer cell line expressing high levels of this polymorphic glycoprotein and whether this regulation could affect metastasis.

## MATERIALS AND METHODS

All media and supplements were purchased from Biological Industries (Beit Haemek, Israel) and all biochemicals from Sigma Chemical Co. (St. Louis, MO, USA) unless otherwise specified.

### Cell line, culture conditions and treatments

HM7 is a cell variant of LS174T, a well-differentiated human colonic adenocarcinoma previously shown to be highly metastatic *in vitro* (Schwartz *et al*, 1992) and *in vivo* (Bresalier *et al*, 1995). Cells were treated with butyrate (2 mM) for 9 days to induce terminal differentiation as described (Schwartz *et al*, 1995a). Cells were treated with 100 mM of the stable phosphorthioate CD44v6-antisense oligonucleotide (5′-CTTCCGTTGTACTACTAGGA-3′) or missense control oligonucleotide (5′-TTTTTTAAAAGGGGCCCCCT-3′). Oligonucleotides were prepared and supplied by General Biotechnology (Rehovot, Israel).

### Western blot analysis

One hundred micrograms cell protein lysates were separated in 10% SDS–PAGE and transferred to a nylon-transfer membrane (Amersham, Buckinghamshire, UK). A positive control protein supplied by the company was included in each analysis. The CD44 protein was reacted with mouse monoclonal anti-human CD44v6 antibody (VFF-18 clone, BMS125, Bender MedSystems, Austria) and visualised by enhanced chemiluminescence kit (ECL, Amersham).

### Immunocytochemistry

Cells were grown in 8-well chamber slides, fixed and immunostained with a 1 : 140 dilution of the anti-CD44v6 monoclonal antibody (V6-B3 clone, Zymed, San Francisco, CA, USA) in blocking buffer. Detection was performed with Vectastain Elit kit Vector Laboratories (Burlingame, CA, USA). Counterstaining was performed with haematoxylin (Schwartz *et al*, 1995b).

### Nuclear run-off

HM7 cells were lysed and their nuclei isolated. ATP, CTP, GTP (2 μM each), UTP (0.04 μM ) and 4 μl of [γ-^32^P]UTP (from 3000 μCi) (NEN™ Life Science Products, Boston, MA, USA) were added to the nuclear suspension (100 μl) and incubated for 30 min. The samples were extracted with phenol/chloroform and precipitated. Five micrograms of the following cDNA probes were slot-blotted onto a nylon transfer membrane (Amersham) and hybridised with the precipitated samples at equal counts per min per ml in hybridisation buffer at 45°C for 48 h: CD44v6 (5′-TGGCATGAGGGATATCGCCAAACACCCAGA-3′) CD44s: (5′-TCTGGGTGTTTGGCGATATCCCTCATGCCA-3′) and β-actin: (5′-GCATGGGTCAGAAGGATTCC-3′). The filters were washed, and the radioactive signals on the autoradiograms were measured using a Fujix Phosphor-Imager apparatus and a BAS-1000 Bio-Imaging Analyzer (Fuji Co., Japan), or exposed to X-ray film.

### CD44 reverse transcription-polymerase chain reaction(RT-PCR)

cDNA was synthesised from 1 to 5 μg of total RNA from control, antisense- or butyrate-treated cells, using an RT–PCR kit (A1250 Promega, Madison, WI, USA). RT–PCR was performed with the prevously reported primers and under similar conditions (Gotley *et al*, 1996): forward primers from exon 10 **(10F)**: 5′-TCCAGGCAACTCCTAGTAGTACAA-3′, or exon 3 **(3F)**: 5′-′TCCCAGTATGACACATATTGC-3′, and reverse primers from exon 17 **(17R)**: 5′-CCAAGATGATCAGCCATTCTGG-3′ or exon 11 **(11R)**: 5′-GATGGCTGGTATGAGCTGAGGCTG-3′.

### Sequence analysis

Sequence analysis of the CD44 variants' (RT–PCR products') cDNA was performed with an automatic thermocyclin sequencing 3700 DNA analyzer ABI prism (Perkin Elmer, Forester City, CA, USA).

### Fluorescence-activated cell sorter (FACS) analysis

Cells were incubated with saturating amounts of anti-CD44v6 or anti-CD44s antibodies (H-CAM, clone F10-44-2, Serotec, Raleigh, NC, USA) and then exposed to FITC (F (ab′) fragment goat anti-mouse IgG (H+L)) (Jackson Immuno-Research Laboratories, Inc, West Grove, PA, USA) antibody and analysed by FACS (FACSort, Becton Dickinson, San Jose, CA, USA).

### Plasmid constructs, DNA transfection and luciferase assay

A fragment of the 5′-flanking region of the human gene encoding CD44 was amplified using the PromoterFinder Kit (CLONTECH, Palo Alto, CA, USA). Primers were designed according to the published human CD44 sequence promoter (Shtivelman and Bishop, 1991). A CD44-specific primer and a nested primer were used in the primary and secondary PCR's, to amplify an 1100-bp fragment from human genomic DNA, spanning part of the promoter sequence and the flanking 100 bp of the 5′-sequence of the CD44 gene. The PCR fragment was subcloned into pSP72 (Promega) and automatically sequenced as described. The nested PCR showed that the promoter sequence is similar to that published previously (Shtivelman and Bishop, 1991) and includes the ERE motif between −604 and −583, as described (Shtivelman and Bishop, 1991; Zhang *et al*, 1997). The resultant 1100 bp fragment was subcloned upstream of the luciferase reporter plasmid pGL3-Basic which contains the luciferase gene to generate pCD44(−1000/+100)-luc. This construct was used to generate point mutations of the CD44 promoter as described in the next paragraph.

### Site-directed mutagenesis

Specific nucleotides in the 1100 bp CD44 promoter were mutated or deleted using the QuickChange Mutagenesis kit (Stratagene, La Jolla, CA, USA). Briefly, a pair of primers each containing two-base-mutated oligonucleotides (from T to C (5′-CCCTCTC**C**CCAGCTCC**C**CTCCC-3′) or from C to A (5′-CCCTCT**A**TCCAGCTCCT**A**TCCC-3′)) was used to mutate the ERE-binding site (−604/−583). The underlined nucleotides were introduced into the mutant constructs, using the QuickChange Mutagenesis kit. The plasmids resulting from the two substitutions were isolated and the mutations confirmed by digestion and by DNA sequencing.

### Transfection studies

HM7 cells were transfected with plasmid DNA (0.5 μg containing the CD44 promoter construct or one of the mutants) and 1.5 μl of FuGENE-6 transfection reagent (Roche Molecular Biochemicals, Indianapolis, IN, USA) for 3 h. The transfected cells were treated for 48 h with EGF (10 nM), butyrate (2 mM) or combinations (incubation time shown to exert significant effects), harvested by centrifugation and washed with 1×PBS, pH 7.4. Cell pellets were lysed with 150 μl of 1×Cell Culture Lysis Reagent (Promega). Following 2 min centrifugation, luciferase activity and protein content of the extracts were measured. For luciferase activity, 10 μl of the extracts were used to measure the integrated light units over 10 s, using the luciferase assay system (Promega), as recommended by the manufacturers.

### Gel shift mobility assay

Nuclear proteins were extracted from 5×10^6^–10^7^ control or butyrate-treated cells as previously described (Andrews and Faller, 1991). Synthetic oligonucleotides of the putative ERE sequence (Zhang *et al*, 1997) and sequences from the mutated ERE oligonucleotides (5′-CCCTCTC**C**CCAGCTCC**C**CTCCC-3′) OR (5′-CCCTCT**A**TCCAGCTCCT**A**TCCC-3′) were annealed and 5′-end-labelled with γ-^32^P-ATP by T4 kinase (BioLab New England, Beverly, MA, USA). Each probe (2×10^4 ^c.p.m.) was applied at a 1 : 1 ratio of premix gel shift reaction in the presence of 10 μg of nuclear extract on ice for 1 h. Poly(dI-dC) (0.2–2 μg) or 10–100 ng unlabelled ERE probe were pre-incubated with the nuclear extracts on ice for 20 min before being reacted with the labelled ERE probe. These unlabelled sequences (Poly(dI-dC) and ERE) served as competition controls. DNA-protein complexes were resolved on a native 4% nondenaturing polyacrylamide gel subsequently dried and autoradiographed (Carey, 1991).

### Liver colonisation

The ability of control, butyrate-, antisense- and missense-treated cells to colonise the liver of athymic mice (Balb/c) was tested as previously described (Bresalier *et al*, 1995). The Animal Welfare Committee of the Faculty of Agricultural, Food and Environmental Quality Sciences, Hebrew University of Jerusalem supervised the research protocols and animal care.

### Statistical analysis

Results were expressed as mean±s.e.m. Statistical significance was calculated by Student's *t*-test.

## RESULTS

### Butyrate regulates CD44 protein expression

**Figure 1:**
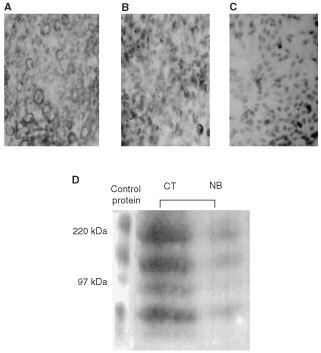
Expression of CD44v6 in HM7 cells. Immunocytochemistry: (**A**) (Magnification (M): ×40): HM7 cells strongly expressed CD44v6. (**B**) (M: ×40): In Butyrate-treated HM7 cells a reduced expression of CD44v6 is observed. (**C**) (M: ×40): Addition of the control protein concomitantly with the anti-CD44v6 antibody almost completely abrogated the binding capacity of the antibody to HM7 cellular antigens. Western immunoblotting: (**D**): The CD44v6 antibody distinguished CD44v6 isoforms within the range of 97–220 kDa either in the control protein as well as in HM7 cells incubated under control conditions (CT) or in butyrate-treated HM7 cells (NB). Representative results from four similar experiments.

HM7 control cells (Figure 1A

), markedly expressed the CD44v6 variant on their surface membrane. Butyrate significantly downregulated CD44v6 expression in HM7 cells after 9 days of treatment (Figure 1B). To demonstrate specificity of the antibody we incubated the sections with both antibody and control peptide. The control protein almost completely abrogated the capability of the antibody to bind cellular antigens. Western immunoblotting with monoclonal antibody to the v6 splice variant revealed expression of various CD44v6 protein isoforms within the range of 97–220 kDa in control cells, similarly to the lanes obtained with the control peptide. Butyrate treatment induced significant downregulation of all CD44v6 isoforms, consistent with the immunostaining results (Figure 1C).

### Butyrate regulates CD44 gene expression

**Figure 2:**
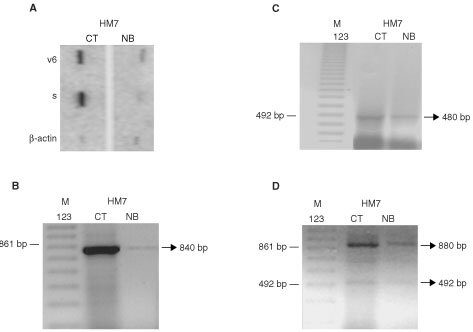
CD44 gene transcription. Nuclear run-off analysis of CD44s and CD44v6 in control HM7 cells (CT) or treated with butyrate (NB) (**A**). HM7 cells transcribed the CD44v6 splice variant. The same results were found for the constant isoform (CD44s). Butyrate inhibited the transcription of both isoforms. RNA loading was verified with β-actin probe. Effect of butyrate treatment on the expression of different splice variants was measured by RT–PCR analysis using primers 10F/17R (**B**), 3F/11R (**C**) and 3F/17R (**D**). Similar results were obtained in 3–5 different experiments.

Nuclear run-off analyses showed that transcription of both the constant and variant species of CD44 was reduced after butyrate treatment (Figure 2A

). RT–PCR analysis allowed detection of alternative splice variants using specific primers to detect CD44 mRNA containing exon 10 (v6) or primers to the borders of the CD44 constant region, in order to detect transcription of the constant region (CD44s) and simultaneously of the larger splice variants of CD44, including several exons of the variant region: (i) RT–PCR assay using the 10F/17R set of primers resulted in a single, evident PCR product of ∼840 bp (Figure 2B). Sequence analysis demonstrated that this band includes all variable exon regions from 10 to 16 and the exon 17 border and (ii) amplification with the set of primers 3F/11R resulted in a single product, demonstrated by sequence analysis to include exons 3, 4, 5a, 10 and the border of exon 11 to give a PCR product of ∼480 bp (Figure 2C). Transcription of the constant CD44 isoform was obtained using the 3F/17R primers for both cell lines. In addition, a number of additional CD44 variants of various sizes were detected with this primer set (Figure 2D). The smallest detectable band was at 492 bp and included exons 3, 4, 5a and 16 (non-variable exons), and the border of exon 17. The second-most notable product was at ∼880 bp, and its sequence analysis revealed the inclusion of exons 3, 4, 5a, 14, 15, 16, and the border of exon 17. Butyrate treatment significantly affected the expression of all CD44 transcripts (Figure 2B–D), suggesting that the short-chain fatty acid regulates the CD44 gene at the transcriptional level.

### Butyrate regulates CD44 promoter activity

**Figure 3:**
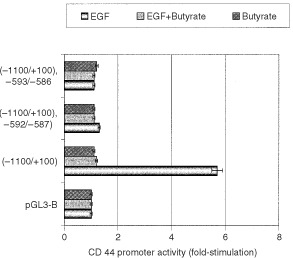
Inducibility of the CD44 promoter. The CD44 promoter linked to a luciferase reporter gene pCD44(−1000/+100)-luc was transfected into HM7 cells. The transfected cells were treated for 48 h with EGF (10 nM), butyrate (2 mM) or combinations. Addition of EGF to the transfectants (−1100/+100) led to a 5.7-fold increase in luciferase activity, compared to cells transfected with pGL3-B. The construct (−1000/+100)−592/−587 is the promoter linked to luciferase containing a T–C mutation at sites −592/−587 and (−1100/+100)−593/−586 is the promoter linked to luciferase containing a C–A mutation at sites −593/−586. Results are mean±s.e.m. of three independent experiments.

A sequence containing a significant portion of the CD44 promoter was generated and linked to pGL3-B which contains the luciferase reporter gene to give the plasmid pCD44(−1000/+100)-luc. Addition of EGF (10 nM) to pCD44(−1000/+100)-luc-transfected HM7 cells for 48 h, led to a 5.5-fold increase in luciferase activity, compared to cells treated transfected with control plasmid, pGL3-B (Figure 3

). Butyrate (2 mM) abrogated luciferase activity induced by EGF in pCD44(−1000/+100)-luc-transfected HM7 cells. Butyrate by its own did not affected luciferase activity in the above-mentioned transfected HM7 cells. To investigate whether the ERE region present in the promoter mediates the induction; substitution-mutated constructs were designed to this specific region in the CD44 promoter. A T–C mutation at sites −592/−587 reduced EGF-induced luciferase transduction by 75%, and a C–A mutation at sites −593/−586 reduced EGF-induced luciferase transduction by 85% (Figure 3), indicating that this ERE site is critical for EGF-induced CD44 gene regulation, and for the down-regulatory effect exerted by butyrate.

### Detection of ERE binding to CD44 promoter

**Figure 4:**
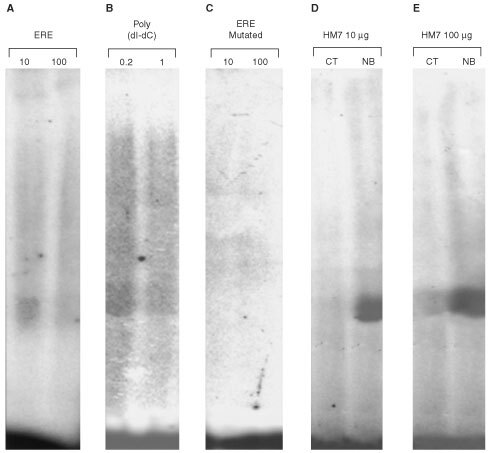
Butyrate-induced protein-ERE complex formation in a gel shift assay. DNA-protein complex formation was measured using ^32^P-labelled 22 bp ERE probe and nuclear extracts of HM7 cells. Competition in protein-DNA formation: 10 and 100 ng unlabelled ERE (**A**), 0.2 and 1 μg poly(dI-dC) (**B**) were incubated with HM7 nuclear extracts before adding labelled ERE probe. Binding specificity determined with 10 and 100 ng ^32^P-labelled C–A replaced ERE probe at the sites indicated by underline (5′-CCCTCT**A**TCCAGCTCCT**A**TCCC-3′) and incubated with nuclear extracts of HM7 cells (**C**, ERE mutated). Nuclear extracts from control (CT) or butyrate-treated (NB) cells were incubated with the labelled ERE probe. The binding was dependent on the concentration of the nuclear protein extracts (**D**; 10 μg, **E**; 100 μg). Representative results from three similar experiments.

To confirm the involvement of ERE in the response to butyrate, electrophoretic mobility shift assays were performed using a labelled 22-bp ERE sequence or site-mutated EREs and nuclear extracts from control or butyrate-treated HM7 cells. Pre-incubation of the labelled ERE with 100 ng unlabelled ERE or 1 μg (dI-dC) reduced the binding of control HM7 nuclear extract to the labelled probe compared to 10 ng of ERE or 0.2 μg of (dI-dC) (Figure 4A,B

). Neither the T–C nor the C-to-A mutated ERE fragments at the indicated sites (see Materials and Methods), bound nuclear extracts of the colon cancer cell line studied (Figure 4C illustrate only the -C–A mutation, the T–C mutation yielded identical results). Butyrate treatment markedly enhanced the formation of protein-DNA complex on the gel, compared to control untreated cells (Figure 4D,E). However, the effect of 100 μg nuclear proteins was most noticeable. These results suggest that the ERE motif located in the CD44 promoter sequence specifically binds nuclear proteins from butyrate-treated cells that may be lacking or exist at very low concentrations in control HM7 cells.

### Effect of antisense treatment on CD44v6 expression

**Figure 5:**
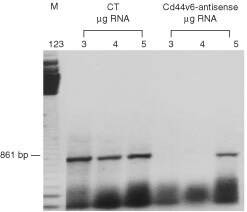
Effect of butyrate and CD44v6-antisense oligonucleotide treatments. CD44v6 isoform including exon 10 was obtained by RT–PCR, using 10F/17R primers in HM7 cells treated with antisense oligonucleotide. Antisense treatment caused a reduction in the mRNA expression of the CD44v6 splice variant. CT: 3, 4 and 5 μg total RNA from control HM7 cells (CT) or CD44v6-antisense treated cells were used in the RT–PCR assays.

We examined the expression of CD44v6 in HM7 cells after treatment with antisense oligonucleotide specific to exon 10 (v6) or missense oligonucleotides by RT–PCR. At lower concentrations of total RNA (3 and 4 μg) the expression of CD44v6 mRNA was not detectable in the antisense-treated cells (Figure 5

). Only at 5 μg of total RNA did expression become evident. In contrast, in control and missense-treated HM7 cells, there was high expression of the variant at all concentrations of total RNA added to the RT–PCR assay.

CD44s and CD44v6 protein expression assessed by FACS analysis show down-regulation of CD44v6 protein expression in HM7 cells treated with CD44v6-antisense compared to control or missense-treated cells. In contrast, the expression of CD44s was barely affected by CD44v6-antisense treatment in HM7 cells relative to control or missense-treated cells. FACS analyses demonstrated that butyrate significantly (*P*<0.001) down-regulates the expressions of both of CD44s and CD44v6, confirming our previous Western and imunocytochemical analyses.

### Effect of CD44 gene regulation on *in vivo* liver colonisation ability of HM7 cells

**Figure 6:**
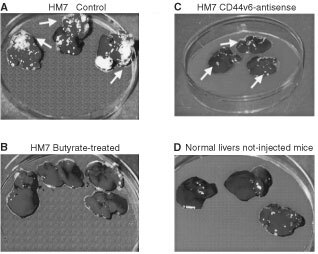
Liver colonisation model. Livers from athymic nude mice 6 weeks after splenic injection of HM7 control cells (**A**), butyrate-treated cells (**B**) CD44v6-antisense treated cells (**C**) compared to non-injected animals (**D**). Results are representative of three independent identical experiments.

**Figure 7:**
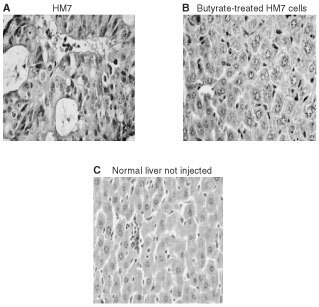
Sections from mouse after liver colonisation assay. H&E staining of liver sections obtained from mice after splenic injection with HM7 cells (**A**), with butyrate-treated HM7 cells (**B**) or normal liver sections from non-injected mice (**C**). Liver tissue and texture was significantly affected following splenic injection with the highly metastatic HM7 cells (**A**).

As expected, control HM7 cells injected into the spleen of athymic Balb/c mice were able to actively colonise the mouse livers, and resulted in significant tumour burden (mean 256±98 nodules per liver, range 49–500, *n*=15,) and the appearance of epitheloid tumours (Figure 6A

see arrows, and Figure 7A

). In HM7 cells treated with butyrate (2 mM) the liver colonisation ability was abolished (Figure 6B). Histology of mouse liver sections showed normal hepatocytes with no infiltration of cells of epithelial origin (Figure 7B). HM7 cells treated with CD44v6-antisense oligonucleotides resulted in a significantly smaller number of micrometastatic foci (26±20 nodules per liver, *P*<0.003). Control non-injected mice exhibited normal livers with no metastatic foci (Figures 6D and 7B).

## DISCUSSION

Functional studies have demonstrated an important role of the expression of CD44 variants and metastasis (Gunther *et al*, 1991; Herrera-Gayol and Jothy, 1999). These observations led us to question whether and how regulation of the expression of CD44 isoforms, including the commonly expressed CD44v6 variant, would affect the metastatic ability of HM7 colon cancer cells. We showed that HM7 cells express high levels of CD44v6, as evidenced by immunocytochemical, Western blot and FACS analyses. RT–PCR analyses with primers to several regions of the CD44 gene were used to characterise the repertoire of CD44 variants expressed in this cell line. We sought whether we could regulate intracellular doses of CD44 protein and its variants by treating the cells with butyrate. We selected this agent because in a recent study (Barshishat *et al*, 2000), we demonstrated that butyrate treatment is able to convert the passive essential promoter of E-cadherin to an active form, thereby affecting gene expression. Butyrate inhibits histone deacetylase leading to histone hyperacetylation (Hennig *et al*, 1995), which can alter chromatin structure and affect accessibility of transcription factors to their recognition sequences, recruit other factors, and control transcription by activation or depression.

In butyrate-treated HM7 cells, CD44v6 protein expression was significantly downregulated. Nuclear run-off and RT–PCR analyses demonstrated that butyrate treatment affect transcription of the entire CD44 gene, not just of specific CD44 variants. RT–PCR analysis confirmed the nuclear run-off findings and showed that any expressed splice variant was drastically downregulated following butyrate treatment.

To establish the mechanism by which butyrate exerts gene transcription repression we created an 1100-bp fragment from human genomic DNA. This sequence spanned part of the promoter sequence and the flanking 100 bp of the 5′-sequence of the CD44 gene (−1000/+100), linked to pGL3-B which contains the luciferase gene to generate pCD44(−1000/+100)-luc. We identified a 22-bp consensus regulatory element to EGF (ERE) within this sequence, between −604 to −583, supporting previous findings (Shtivelman and Bishop, 1991; Zhang *et al*, 1997). Then, two-base-targeted mutations of this sequence at ERE site (−604 and −583) were obtained. The intact (−1000/+100) promoter responded maximally to EGF treatment. Butyrate completely abrogated this EGF-mediated CD44 promoter activity. Butyrate by itself did not affected promoter activity. Mutations targeted to the ERE sequence completely eliminated the luciferase inducibility of the CD44 promoter exerted by EGF, and the respective inhibitory action exerted by butyrate. We then demonstrated that butyrate induces binding of nuclear proteins specifically to ERE since point-mutated ERE constructs did not bind nuclear proteins following butyrate treatment. The specific nuclear proteins induced by butyrate to bind to ERE remain to be identified, but since the motif is related to EGF, it is plausible to assume that they are related to this growth factor.

We next asked whether butyrate treatment affects CD44-mediated biological activity, in an *in vivo* experimental setting. The *in vivo* liver colonisation assay following intrasplenic injection of metastatic HM7 cells into nude mice demonstrate that these cells were able to invade the liver, proliferate and create metastatic tumours. HM7 cells treated with CD44v6-antisense oligonucleotides exhibited a significantly lower capacity to create metastatic nodules in the mouse livers. Remarkably, butyrate-treated HM7 cells completely lost their ability to colonise the livers of nude mice. Inoculation of HM7 cells treated with butyrate (1 mM, data not shown) into nude mice induced a more moderate reduction in liver nodule formation (256±98 in control to 90±5 in butyrate treated, 65% reduction). Inoculation of HM7 cells treated with butyrate (0.1 mM, data not shown) into nude mice induced a more borderline reduction in liver nodule formation (256±98 in control to 190±25 in butyrate treated, 25% reduction). Regarding the time window, at 2 mM concentration 9 days also gave optimal results, while 5 and 7 days of treatment were less effective (not shown). These findings suggest that CD44v6 is an essential variant protein for metastatic cell growth in the host organ (Kaya *et al*, 1997). Reducing the expression of the CD44v6 variant by antisense treatment resulted in significant inhibition of metastatic nodule development. However, pan-deregulation of several other CD44 variants appears to be more effective. In line with these findings, Reeder *et al* (1998) reported that HT29 cells transfected with antisense vectors to CD44 exon 10 and inoculated into nude mice result in mice completely free of metastasis for 16 weeks, as compared to a 30% metastasis rate in the control HT29 parental and vector inoculated mice for the same period. The relatively low incidence of metastasis obtained following inoculation of parental HT29 cells may be explained by the fact that this cell line is not highly metastatic as are HM7 cells, which are able to produce 100% metastasis incidence after only 6 weeks. Harada *et al* (2001) findings further support our study: they show that mice intrasplenically injected with LS174T cell transfectants with antisense CD44s vectors were completely free of metastasis as compared to mice injected with the parental LS174T cells. Transfected cells with antisense CD44s inhibited the overall expression of CD44 variants, an effect that in our study we achieve following treatment with butyrate. Since CD44 variants serve as low-affinity receptors for growth factors (Ponta *et al*, 1998, Sherman *et al*, 1998), reducing CD44 variants expression may result in the prevention of growth factor presentation and in the concomitant inhibition of metastatic tumour development.

Since butyrate controls the expression of additional genes, we cannot exclude the possibility that they might also play a role in the observed dramatic inhibition of metastasis.

We believe that butyrate control the expression of additional genes involved in metastasis. We have shown previously that butyrate upregulates E-cadherin transcription and expression (Barshishat *et al*, 2000), a molecule known to act as tumour suppressor gene in metastasis. In addition, a recent publication by Pelizzaro *et al* (2002), show that butyrate induced a dose-dependent down-regulation of VEGF165 protein, a potent angiogenic factor and vis-à-vis up-regulates the hypoxia-inducible factor (HIF)-1α. Our reported effects on CD44 and the effects reported by our group previously (Barshishat *et al*, 2000) and by Pelizarro *et al* (2002) may induce altogether the significant effects induced by butyrate observed *in vivo* on metastatic formation. Nevertheless, the pan-deregulatory effect exerted by butyrate in the entire CD44 gene seems to play a key role in the complete abolishment of metastatic ability exhibited by HM7 cells. CD44 variants bind to a wide variety of ECM constituents and growth factors which have been shown previously to play different roles in tumour invasion, motility and metastasis (Katagiri *et al*, 1999), and downregulation of their expression may be pivotal for the metastatic cascade to take place.
